# Nitrogen-doped Carbon Dots Mediated Fluorescent on-off Assay for Rapid and Highly Sensitive Pyrophosphate and Alkaline Phosphatase Detection

**DOI:** 10.1038/s41598-017-06356-z

**Published:** 2017-07-19

**Authors:** Yalei Hu, Xin Geng, Lin Zhang, Zhongming Huang, Jia Ge, Zhaohui Li

**Affiliations:** 1 College of Chemistry and Molecular Engineering, Zhengzhou University, Zhengzhou, 450001 P.R. China; 2 Institute of Chemical Biology and Nanomedicine, Hunan University, Changsha, 410082 P.R. China

## Abstract

In this report, a novel fluorescent sensing platform using nitrogen-doped carbon dots (N-CDs) as probes for fluorescence signal transmission has been designed for the detection of significant biomolecules pyrophosphate (PPi) and alkaline phosphatase (ALP). The high fluorescent N-CDs could be selectively quenched by Cu^2+^, and recovered by the addition of PPi because PPi preferentially binds to Cu^2+^. Once ALP was introduced into the system, ALP can specifically hydrolyze PPi into Pi, the intense fluorescence of N-CDs could be quenched again due to the recombination of the as-released Cu^2+^ with N-CDs. So, fluorescence of N-CDs is regulated by an ALP-triggered reaction. Based on this strategy, we demonstrated that N-CDs could serve as a very effective fluorescent sensing platform for label-free, sensitive and selective detection of PPi and ALP with low detection limit of 0.16 μM and 0.4 U/L for PPi and ALP, respectively. Moreover, the assay time is just around 0.5 min for PPi and 30 min for ALP. This developed strategy shows remarkable advantages including sensitive, rapid, simple, convenient, and low-cost and so forth. Furthermore, this method was also successfully applied to monitor ALP in human serum, which indicates its great potential for practical applications in biological and clinical diagnosis.

## Introduction

Pyrophosphate (PPi), formed by a condensation reaction of two inorganic phosphate units, plays a key role in energy transduction and several major metabolic processes^1, 2^. For example, PPi concentration can indicate pivotal information such as DNA replication, which can be used in cancer diagnosis by monitoring telomerase elongation process^3^. Alkaline phosphatase (ALP), as a membrane-bound enzyme, is one of the most commonly used hydrolase enzyme found in various sources of mammals (bone, liver, placental, and intestinal), which has been widely utilized as an important biomarker for clinical diagnostics^4, 5^. The abnormal level of ALP in the human body is a signal for a variety of diseases states, particularly involving in the liver, prostate and the bone^6–9^. Moreover, ALP is also used as a marker reagent for biological studies. Therefore, the accurate determination of ALP and PPi is essential in biochemical study and clinic diagnosis.

Up to date, many techniques have been developed for the detection of PPi and ALP including colorimetric^10, 11^, fluorometric^12, 13^, surface-enhanced Raman scattering^14^ and electrochemical methods^15, 16^. Among these assays, fluorometric methods have attracted considerable interest for their rapid response, easy operation and high sensitivity^17–19^. Traditional fluorescent methods are mainly based on organic dyes^20, 21^, fluorescent polymers^22, 23^ and metal nanoclusters or nanoparticles^24–26^. Whereas, most of them have poor photostability, laborious synthetic procedures, and complexed labeling processes. For instance, an organic fluorescent probe HCAP has been reported for ALP activity detection with aggregation-induced emission (AIE)^27^. However, like most of organic dyes, HCAP has poor photostability and solubility in aqueous system, which limits its further practical application. Jia *et al*. reported fabrication of β-cyclodextrin-modified CdTe quantum dots for ALP detection via electron transfer^28^. Nevertheless, the high toxicity and environmental hazards owing to the heavy metal Cd^2+^ have intrinsically confined its further application. Very recently, a DNA-scaffold silver nanocluster was constructed as the fluorophore to detect PPi and ALP activity with the assistance of copper ion^29^. However, synthesis of noble metal nanoclusters requires high cost and arduous synthetic procedures; also, they have poor optical and chemical stability in aqueous system. Consequently, there is still an urgent demand for developing a simple, cost effective, sensitive and rapid method for PPi and ALP activity detection.

Fluorescent carbon dots (CDs), as a new class of carbon-based fluorescent nanomaterials with size below 10 nm, have attracted increasing attention due to their superiorities in biocompatibility, photostability, aqueous solubility and tunable fluorescent properties compared with organic dyes and semiconductor quantum dots^30–32^. In view of the outstanding properties, CDs have been widely used in bioimaging, biosensing, photocatalysis and drug/gene delivery^33–38^. However, only a few fluorescent assays based on CDs have been reported for PPi or ALP activity detection. Qian’s group^39^ synthesized CDs for ALP detection based on aggregation/disaggregation of CDs. Although the fluorescence displayed reasonable sensitivity, lengthy preparation process and extremely low fluorescence quantum yield (2.2%) of the CDs limited its further biomedical application. Recently, studies have shown that heteroatom doping, especially nitrogen doping, could be used to fine-tune or obtain new kinds of high-performance CDs^40^, which hold great potential for biosensing and bioanalysis studies.

Herein in this report, we present an on-off switch fluorescent assay for PPi and ALP activity detection by using highly fluorescent N-CDs as signal transducer. The N-CDs were prepared by a one-step and green solid phase method using sodium alginate and tryptophan as the precursors. The fluorescence of N-CDs could be quenched by Cu^2+^, and recovered by the addition of PPi. ALP could specifically hydrolyze PPi into Pi, the intense fluorescence of N-CDs could be quenched again due to the recombination of the as-released Cu^2+^ and N-CDs. Experimental results demonstrate that this proposed assay has robust ability for quantitative analysis of both PPi and ALP activity with high sensitivity, low cost, good selectivity and rapidity as well as simplicity, which are highly desired for the screening of PPi and ALP in clinical diagnostics and other biomedical applications.

## Results

### Structural and morphological properties of N-CDs

**Figure 1:**
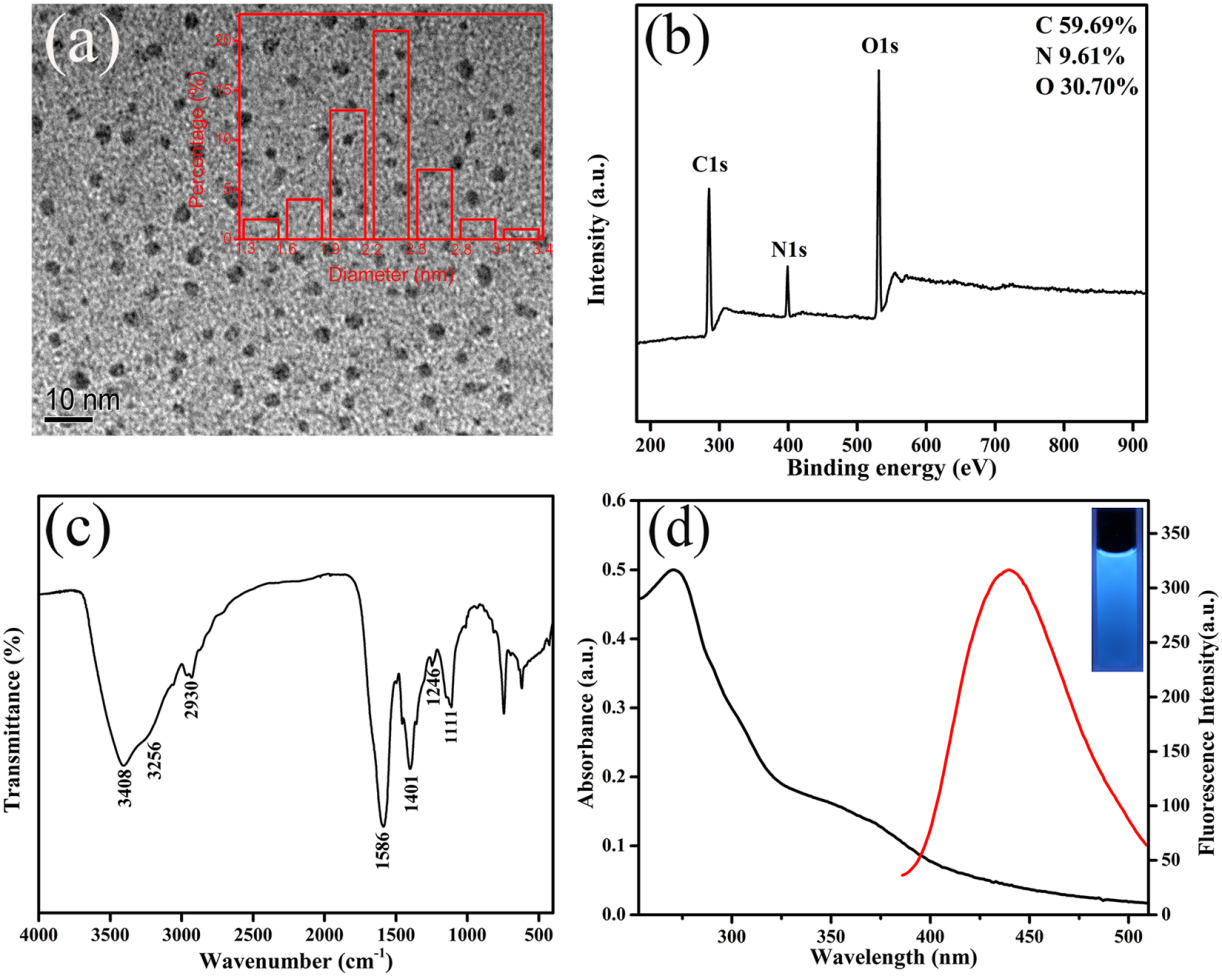
(**a**) TEM image of N-CDs. Inset: Corresponding size distribution histograms of N-CDs. (**b**) XPS spectrum of N-CDs. (**c**) FT-IR spectrum of N-CDs. (**d**) Absorption and fluorescence emission spectra of N-CDs. Inset: Fluorescence image of N-CDs solutions with UV irradiation.

Transmission electron microscopy (TEM) image in Fig. 1a shows the morphological of N-CDs prepared by solid phase method. The N-CDs are nearly spherical and monodispersed, which have an average diameter about 2.3 nm. In order to investigate the structure and composition of N-CDs, X-ray photoelectron spectroscopy (XPS), Fourier transform infrared (FT-IR) and X-ray diffraction (XRD) were used for further characterization. As shown in Fig. 1b, three strong peaks appeared at 285.3, 399.1, and 531.6 eV, which are associated with C1s, N1s, and O1s, respectively. The XPS spectrum of C1s (Supplementary Fig. S1a) exhibits three fitted peaks at 284.0, 285.5 and 287.1 eV. The binding energy at 284.0 eV could be assigned to the graphitic structure (C–C). The peak at around 285.5 eV suggests the presence of C–O and C–N, and the peak about 287.1 eV is associated with C=O group^41^. The high-resolution spectrum of O1s (Supplementary Fig. S1b) reveals the presence of both C=O group and C–OH/C–O–C group. The deconvolution of the N1s spectrum (Supplementary Fig. S1c) displays two peaks at 398.7 and 399.4 eV, which are associated with N in a pyridnic N and pyrrolic N^42^. In addition, the N/C atomic ratio was calculated to be 16.1%. In the FT-IR analysis of N-CDs, the following were observed: stretching vibration of O–H at 3408 cm^−1^ and C–H at 2930 cm^−1^, stretching vibration and bending vibration of N–H at 3256 cm^−1^ and 1586 cm^−1^, bending vibration of the C–O at 1401 cm^−1^ and the stretching peak of C–O–C at 1111 cm^−1^, C–N stretching vibration at 1246 cm^−1^ (Fig. 1d)^43–45^. These results indicate that there are abundant of hydroxyl, amino and carboxyl group on the surface of the N-CDs. The XRD spectrum (Supplementary Fig. S2) exhibits a broad peak at around 23°, corresponding to a disordered carbon atoms^46^. The above facts suggest that the as-prepared CDs are N-doped and exhibit abundance hydrophilic groups on the surface.

### Optical properties of N-CDs

In order to investigate the optical properties of the N-CDs, UV-vis absorption and fluorescence spectra (Fig. 1d) were recorded, respectively. As shown in Fig. 1d, the as-prepared N-CDs show an narrow absorption peak at 272 nm and a weak shoulder at around 370 nm (black curve), which are attributed to the π-π* transition of aromatic sp^2^ domains and n-π* transition of C=O bond, respectively^47, 48^. Meanwhile, the peak emission of N-CDs occurs at 440 nm with the maximum excitation at 272 nm (red curve). A strong blue fluorescence could be observed when N-CDs aqueous solution was placed under a hand-held UV lamp (inset in Fig. 1d). As shown in Supplementary Fig. S3, the emission wavelength shows nearly no shift when the excitation wavelength is changed from 250 nm to 410 nm, indicating that the N-CDs exhibit excitation-independent PL behavior, which is considered to be related to less surface defects and more uniform size. The absolute fluorescence quantum yield (QY) of the as-prepared N-CDs was detected to be 43.2% by using a FLS 980 fluorometer equipped with an integrating sphere (IS)-based absolute QY measurement system. Also, the N-CDs are very stable, whether stand several months at room temperature (Supplementary Fig. S4a) or under 272 nm light illumination for 1 hour (Supplementary Fig. S4b), the fluorescence signal almost remains constant, which facilitates their further application. Moreover, the fluorescence lifetime of N-CDs was measured to be 14.10 ns according to time-correlated single-photon counting technique, and the result was shown in Supplementary Fig. S5.

### Design and construction of assay

**Figure 2:**
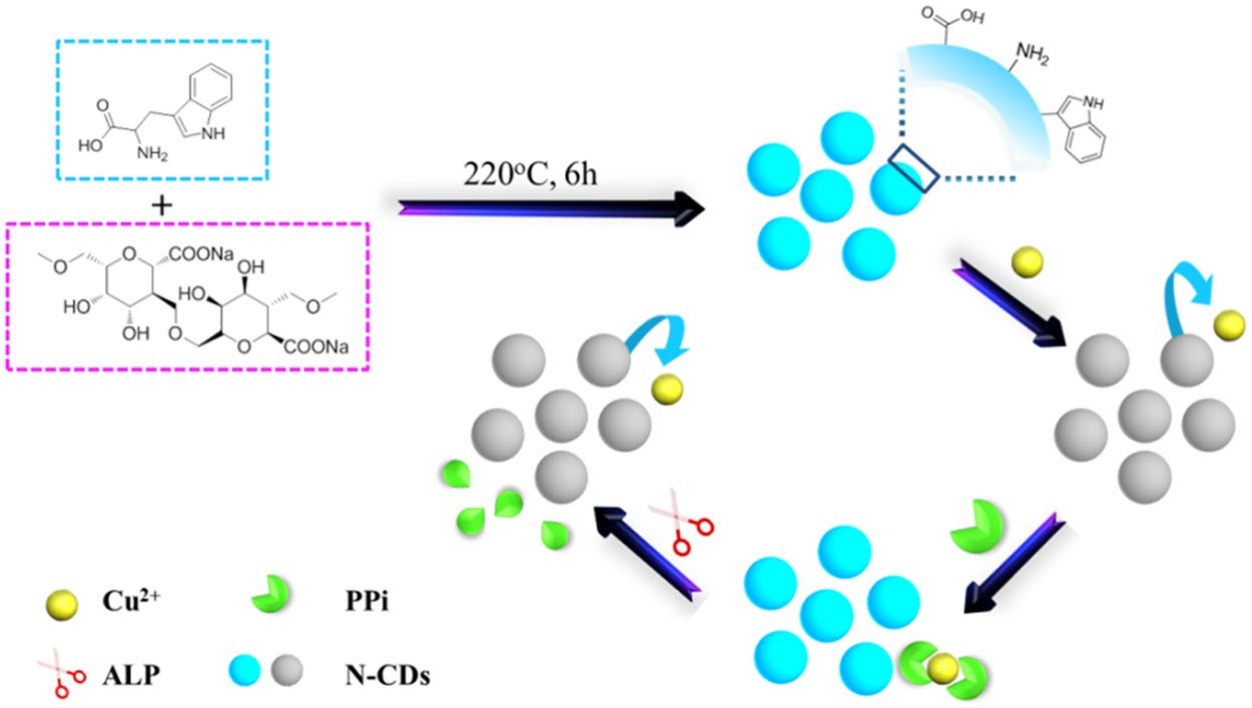
Schematic illustration of the detection strategy for PPi and ALP activity by using N-CDs.

As illustrated in Fig. 2, the designed strategy for PPi and ALP activity assay is based on N-doped carbon dots (N-CDs). Typically, the as-prepared N-CDs with strong blue fluorescence can be selectively and sufficiently quenched by Cu^2+^, and recovered immediately after the addition of PPi due to much higher stability constant between PPi and Cu^2+^. Once ALP is introduced into the sensing system, PPi would be hydrolyzed into Pi fragments. Consequently, the fluorescence of N-CDs is quenched again because of the reintegration of N-CDs with the as-released free Cu^2+^. Based on this concept, quantitative evaluation of PPi as well as ALP activity could be realized very easily by using a single sensing system.

**Figure 3:**
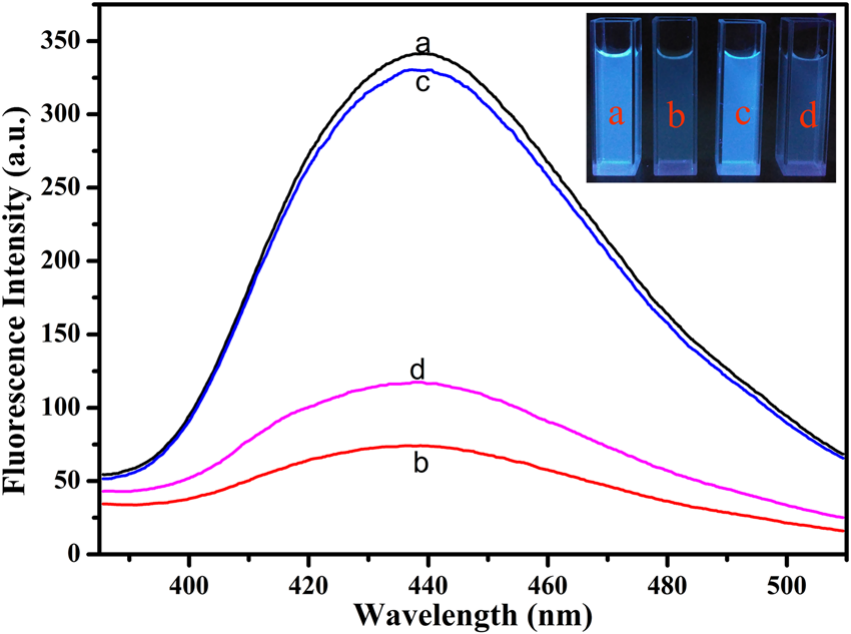
Fluorescence spectra and image of N-CDs in the presence of different composition (**a**) mere N-CDs; (**b**) in the presence of 5 μM Cu^2+^; (**c**) in the presence of 5 μM Cu^2+^ and 50 μM PPi; (**d**) in the presence of 5 μM Cu^2+^, 50 μM PPi and 1 U/mL ALP.

The corresponding responses of this sensing system at different stages were shown in Fig. 3. In the presence of Cu^2+^, the as-prepared N-CDs with strong fluorescence (curve a) were efficiently quenched (curve b), suggesting that an effective electron or energy transfer process have happened between Cu^2+^ and N-CDs^49, 50^. This transfer process was mainly caused by the chelation of Cu^2+^ with N-CDs, which could be ascribed to the quite high thermodynamics affinity of Cu^2+^ for typical N,O-chelate ligands on the surface of the N-CDs and rapid metal-to-ligand binding kinetics^51, 52^. In order to investigate the driving force between Cu^2+^ and CDs, Zeta potential measurement has been carried out. As shown in Supplementary Fig. S6, the Zeta potential of the N-CDs and copper ions is −27.85 ± 1.5 mV and 0.49 ± 0.5 mV, respectively. When Cu^2+^ is added into the CDs solution, the Zeta potential becomes to be −13.88 ± 1.2 mV, suggesting that the driving force between CDs and Cu^2+^ is electrostatic adsorption. Once PPi was added into the N-CDs/Cu^2+^ solution, a clear recovery of the fluorescence (curve c) of N-CDs was observed because PPi preferentially bound to Cu^2+^ with very high stability constant of PPi-Cu^2+^ complex (stability constant log *K*_PPi-Cu_ = 12.45)^53^. After ALP was introduced into the system, PPi was hydrolyzed to Pi and the fluorescence of N-CDs was quenched again (curve d) as a result of the reintegration of N-CDs and the as-released Cu^2+^. The results indicate the great possibility of this N-CDs-based sensing platform for sensitive PPi and ALP detections.

### Detection of PPi

We firstly investigated the effect of Cu^2+^ concentration in this sensing system. As shown in Supplementary Fig. S7, the fluorescence intensity of N-CDs decreases gradually with increasing Cu^2+^ concentrations. When the concentration of Cu^2+^ is higher than 5 μM, the quenching efficiency slows down and then reaches to equilibrium. Thus, 5 μM was selected as the optimal concentration for the following experiments. Meanwhile, the quenching time and selectivity were also studied. As shown in Supplementary Fig. S8a, with only 5 μM Cu^2+^, the efficiency of fluorescence quenching was up to about 80% in just 30 seconds. And as shown in Supplementary Fig. S8b, Cu^2+^ can selectively quench the fluorescence of N-CDs.

**Figure 4:**
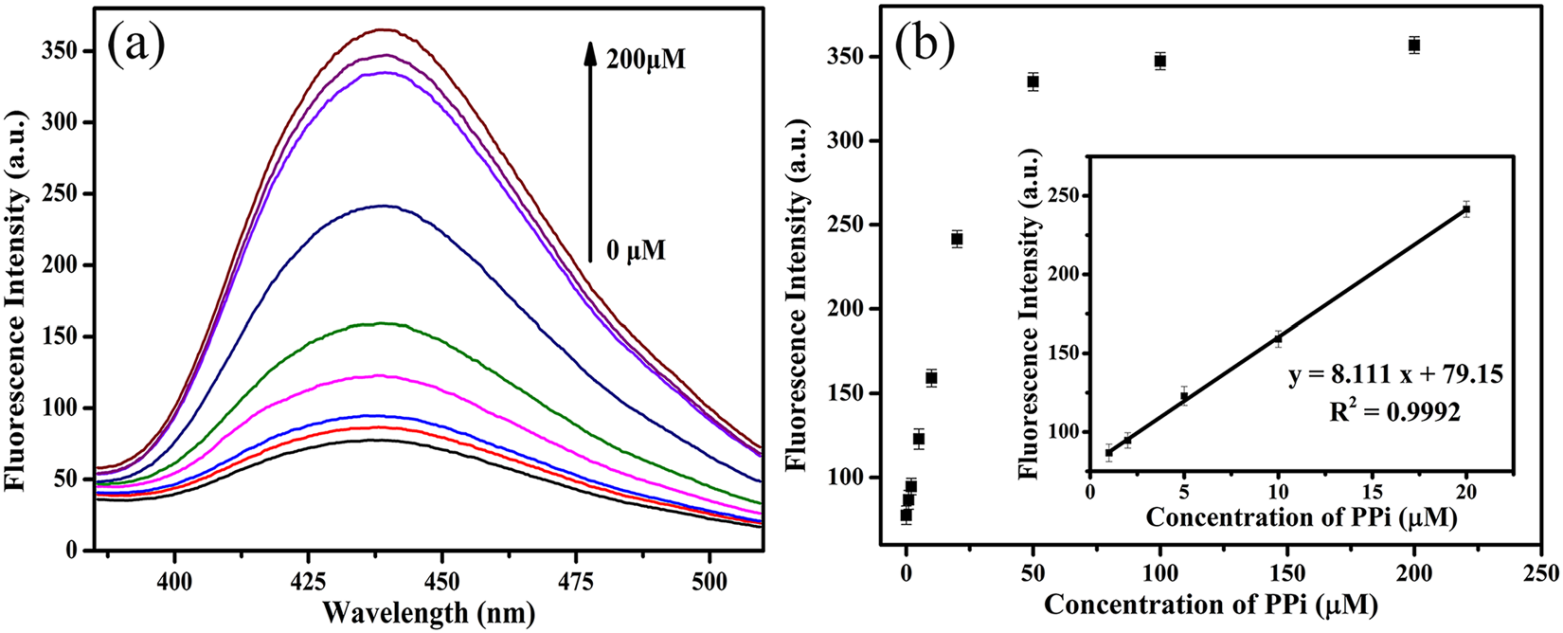
(**a**) Fluorescence spectra of the mixture containing N-CDs, Cu^2+^ and increasing amount of PPi (0, 1, 2, 5, 10, 20, 50, 100, 200 μM). (**b**) The fluorescence intensity of the mixture versus the concentration of PPi. Inset: The fitting curve between fluorescence intensity and PPi concentration.

The quenched fluorescence of N-CDs by Cu^2+^ could be rapidly recovered once PPi was introduced into the system. As can be seen from Supplementary Fig. S9a, the fluorescence intensity of the mixture recovers quickly to almost 100% when the PPi concentration is 50 μM after an incubation time of only 30 seconds, which indicates that this assay is a very rapid method for PPi detection. The effect of reaction pH values was also optimized, and the results revealed that fluorescence intensity has a slight difference in a buffer solution of pH 6.5–8.5 (Supplementary Fig. S10). These results indicate that this proposed strategy could be used for the detection of PPi when the pH values range from 6.5 to 8.5 and pH 7.4 was used as the experimental condition due to the relatively higher fluorescence recovery. Under the above optimized experimental conditions, the fluorescence intensity of N-CDs was collected after adding different concentrations of PPi from 0 to 200 μM into the N-CDs/Cu^2+^ mixture for PPi sensing in 10 mM Tris-HCl buffer (pH 7.4) at room temperature. As shown in Fig. 4a, the fluorescence intensity of the N-CDs gradually recovers with the increasing PPi concentrations, and then reaches to maximum when the concentration of PPi is 200 μM. A good linear relationship between the fluorescence intensity and PPi concentration was obtained over the range from 1 to 20 μM (Fig. 4b inset). The linear regression equation is y = 8.111 x + 79.15 (y is the fluorescence intensity of N-CDs/Cu^2+^ mixture in the presence of different concentrations of PPi and x is the concentration of PPi), R^2^ = 0.9992, and the detection limit of the developed assay for PPi is 0.16 μM according to the 3σ rule. Moreover, we further compared the sensitivity and assay time of this approach with other fluorescent methods as shown in Table 1, which are more sensitive or comparable with that obtained by the previous reported strategies. To validate the selectivity of our method for PPi sensing, we further prepared different anions as potential interferents. As shown in Supplementary Fig. S9b, the possible interferents including F^−^, Cl^−^, Br^−^, I^−^, SO_4_^2−^, NO_3_^−^, HCO_3_^−^, Ac^−^, and PO_4_^3−^ cannot affect this competitive assay. The fluorescence responses of N-CDs/Cu^2+^ mixture were also investigated in the presence of sulphur compounds including Cys, GSH, HS^−^ and HSO_3_^−^. As can be seen from Supplementary Fig. S9c, HSO_3_^−^ has little effect on the fluorescence intensity of N-CDs/Cu^2+^ mixture. Because Cys, GSH, and HS^−^ can also bind with Cu^2+^, the fluorescence is enhanced after the addition of Cys, GSH and HS^−^. According to the previous paper, the interference of Cys, GSH and HS^−^ could be eliminated by adding N-ethylmaleimide (NEM, a RSS blocking agent)^54^. So, after incubation of Cys, GSH or HS^−^ with NEM, the addition of N-CDs/Cu^2+^ mixture gives a negligible signal enhancement, which indicates that this proposed strategy could be successfully used for the detection of PPi with the introduction of NEM. These results indicate that this N-CDs-based assay is very fast, highly sensitive and selective for PPi detection.

**Table 1:** Comparison of sensitivity and assay time of our assay with other fluorescent methods for PPi and ALP activity detection.

Material	Analyte	LOD	Assay time	Reference
Carbon quantum dot	PPi	2.56 μM	2 min	12
Silver nanocluster	PPi	0.11 μM	65 min	29
Spiropyran compound	PPi	0.4 μM	5 min	13
Gold nanocluster	PPi	2 μM	2 min	25
Carbon quantum dot	PPi	0.3 μM	3 min	9
AuNR@SiO_2_@TCPP	PPi	0.82 μM	10 min	1
N-CDs	PPi	0.16 μM	0.5 min	This work
CdTe quantum dots	ALP	10 U/L	30 min	28
Copper nanoparticles	ALP	0.3 U/L	60 min	26
Carbon quantum dot	ALP	1.4 U/L	60 min	39
Silver nanocluster	ALP	5 U/L	130 min	29
Carbon quantum dot	ALP	1.1 U/L	30 min	9
Gold nanocluster	ALP	0.1 U/L	90 min	25
N-CDs	ALP	0.4 U/L	30 min	This work

### Detection of ALP

**Figure 5:**
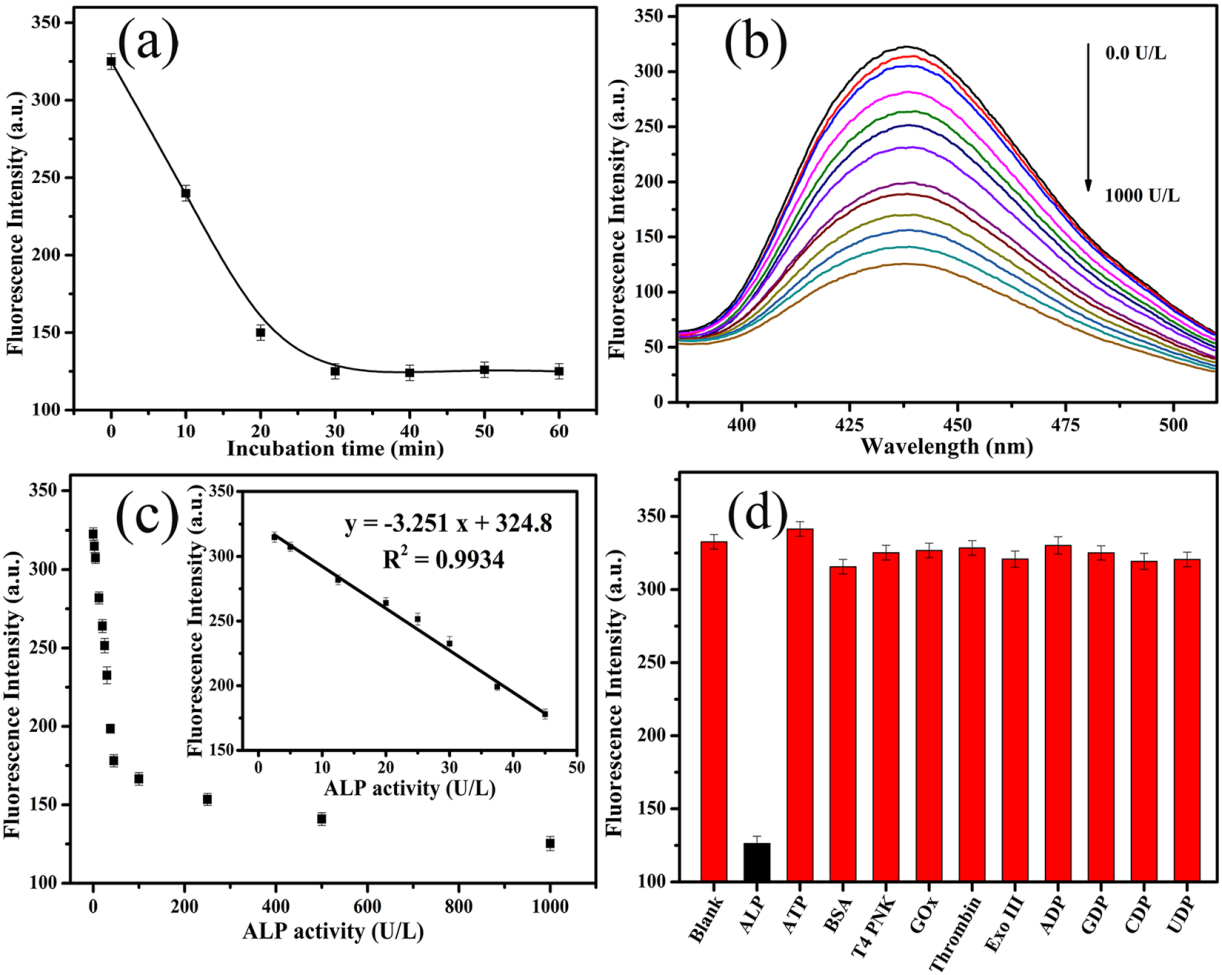
(**a**) The fluorescence intensity of the mixture containing N-CDs, Cu^2+^ (5 μM), PPi (50 μM) and ALP (1 U/mL) versus the incubation time. (**b**) The fluorescence emission spectra of the mixture containing N-CDs, Cu^2+^ (5 μM) and PPi (50 μM) as increasing ALP concentration (0, 2.5, 5, 12.5, 20, 25, 30, 37.5, 45, 100, 250, 500 and 1000 U/L). (**c**) The fluorescence intensity of the mixture versus ALP concentration. Inset: The fitting curve between fluorescence intensity and ALP concentration. (**d**) Selectivity investigation of the proposed assay for ALP activity detection. The concentration is 0.15 μg/mL for ALP and 10 μg/mL for other targets.

The activity of ALP was monitored by coupling with the N-CDs/Cu^2+^-PPi system in 10 mM Tris-HCl buffer (pH 7.4). We first investigated the effect of the incubation time on the ALP assay. As shown in Fig. 5a, the fluorescence intensity of the mixture gradually decreases with increasing reaction time. Considering the quenching efficiency and time consumption, 30 min was selected as the appropriate time for the following experiments. The fluorescence spectra of the system after incubating with different concentrations of ALP (0–1000 U/L) was shown in Fig. 5b. Meanwhile, a good linear relationship between the fluorescence intensity and ALP activity was obtained over the range from 2.5 to 45 U/L (Fig. 5c inset). The linear regression equation is y = −3.417 x + 324.6 (y is the fluorescence intensity of N-CDs/Cu^2+^-PPi system in the presence of different concentrations of ALP and x is the concentration of ALP), R^2^ = 0.9913. The detection limit of 0.4 U/L was achieved according to the 3σ rule, which is sensitive enough for ALP activity assay in biological samples (46–190 U/L for adults)^55^. The sensitivity and assay time of this approach were also compared with other fluorescent methods. As can be seen from Table 1, our method shows either comparable or even better response. To explore the specificity of this proposed approach for ALP activity detection, we prepared several different possible interferents including adenosine triphosphate (ATP), bovine serum albumin (BSA), T4 Polynucleotide Kinase (T4PNK), glucose oxidase (GOx), thrombin, exonuclease III (Exo III) and NDP (ADP, GDP, CDP and UDP). As shown in Fig. 5d, only ALP leads to an obvious decrease of fluorescence while the others showed little interference on the response of this bioassay to ALP. These results clearly demonstrate that this proposed assay is highly sensitive and selective for ALP activity detection.

### Detection of ALP in human serum samples

To demonstrate the feasibility of this procedure in real serum samples, we investigated the analytical performance of this assay for ALP sensing in diluted human serum (1%). Under optimized experimental conditions, we added varying concentrations of ALP into the N-CDs/Cu^2+^-PPi system in Tris-HCl buffer solution (pH 7.4) containing 1% diluted human serum, and the fluorescence signals were collected after 30 min incubation at 37 °C. As shown in Supplementary Fig. S11, a good work linear equation for ALP activity sensing in serum was obtained over the range from 5 to 100 U/L. With the regression equation in serum, three serum samples with the addition of different concentrations of ALP were measured. As shown in Supplementary Table S1, satisfactory recoveries between 96.0% and 104.0% are achieved with relative standard deviation (RSD) below 1.9%. Successfully detecting the human serum samples displays the promise of the present method for ALP analysis with good accuracy and reliability in various clinical applications and biological studies.

In conclusion, we have developed a rapid N-CDs mediated fluorescent on-off assay for highly sensitive and selective PPi and ALP activity detection. The N-CDs were prepared facilely by a one-step and green solid phase method. The assay relies on the principle that the fluorescence of N-CDs could be efficiently quenched by Cu^2+^, and recovered by the addition of PPi, which removes Cu^2+^ from the surface of N-CDs. Once ALP was introduced into the system, PPi was hydrolyzed into Pi and the fluorescence of N-CDs could be quenched again due to the recombination of N-CDs and Cu^2+^. Under the optimized experimental conditions, the assay exhibits high selectivity and sensitivity for PPi and ALP activity detection with very low detection limit of 0.16 μM and 0.4 U/L, respectively. Moreover, we have also applied this assay to monitor ALP activity in human serum and achieved satisfactory results successfully. Taking full advantages of N-CDs, this assay exhibits significantly properties including good sensitivity and selectivity, short assay time, simple design, convenient operation, low cost and environmental friendliness, which promise its great prospect for practical application in biological and clinical diagnosis.

## Methods

### Materials

Sodium alginate, tryptophan, and pyrophosphate (PPi) were obtained from Aladdin reagent Co., Ltd. (Shanghai, China). Alkaline phosphatse (ALP) from calf intestinal mucosa, T4 Polynucleotide Kinase (T4 PNK), and exonuclease III (Exo III) were purchased from New England biolabs (Ipswich, MA). Adenosine triphosphate (ATP), bovine serum albumin (BSA), glucose oxidase (GOx), and thrombin were purchased from Sigma-Aldrich (St. Louis, MO, USA). All reagents of analytical grade were directly used without additional purification. The reaction buffer solution employed in this work was 10 mM Tris-HCl (pH 7.4). All solutions were prepared by using ultrapure water which was obtained through a Millipore Milli-Q water purification system (Billerica, MA) with an electric resistance ≥18.2 MΩ.

### Synthesis of N-CDs

The N-CDs were prepared by a green and facile solid phase method according to a previously reported method with minor modification^43^. Briefly, tryptophan (10 mmol, 2.04 g) and sodium alginate (1.08 g) were mixed and ground to a uniform powder in an agate mortar. The prepared mixture was transferred into a Teflon-lined stainless steel autoclave (25 mL) and heated at 220 °C for 6 h. After being cooled down to room temperature, the obtained dark brown product (yield ca. 58%) was dissolved in 20 mL ethanol, and then centrifuged at 11000 rpm for 10 min to remove any precipitations. The supernatant was collected, mixed with 60 mL toluene and centrifuged at 15000 rpm for 10 min. The precipitate was collected, and then dried at 60 °C to obtain the light brownish products of N-CDs.

### Instrumentation

The morphology of N-CDs was characterized by transmission electron microscopy (TEM) (FEI Tecnai G2, USA) at an accelerating voltage of 200 kV. A drop of sample solution was placed on a copper grid that was left to dry before being transferred into the TEM sample chamber. Fluorescence experiments and ultraviolet-visible (UV-vis) measurements were carried on F-4600 spectrophotometer (Hitachi, Japan) and Agilent 8453 UV-vis spectrophotometer (USA), respectively. The fluorescence emission spectra of N-CDs were collected from 385 nm to 510 nm at room temperature with an excitation wavelength at 272 nm. The X-ray photoelectron spectroscopy (XPS) analysis was conducted by an ESCALAB 250Xi instrument (Thermo Fisher Scientific, USA) equipped with Al Kα (1486.6 eV). Fourier transform infrared (FT-IR) spectrum was recorded in the range of 4000–400 cm^−1^ by using a NEXUS-470 spectrometer (Nicolet, USA) with KBr pellets. The Quantum Yield and lifetime of N-CDs were determined by using a FLS 980 fluorescence spectrophotometer (Edinburgh, UK). The Zeta potential measurements were carried out by using a zeta/nano particle analyzer (Nano Plus, Micromeritics Instruments) at room temperature.

### PPi detection procedure

In a typical experiment for PPi detection, the mixture of 20 μL Cu^2+^ (final concentration of 5 μM) and 20 μL PPi of different concentrations were added into Tris-HCl buffer solution (pH 7.4) containing N-CDs (final concentration of 10 μg/mL) in 200 μL centrifuge tube. After incubation for 1 min at room temperature, the fluorescence spectrum was recorded at the excitation wavelength of 272 nm. To examine whether other anions could interfere with the detection of PPi, the specificity assay was performed. The concentration of all other anions was 500 μM in the N-CDs/ Cu^2+^ system, which is 10-fold than that of PPi. All reaction had been performed in triplicate to ensure reproducibility.

### ALP detection procedure

A typical experimental procedure was carried out as follows: different concentrations of 20 μL ALP were added to the Tris-HCl buffer solution (pH 7.4) containing 20 μL N-CDs (final concentration of 10 μg mL^−1^), 20 μL Cu^2+^ (final concentration of 5 μM), 20 μL PPi (final concentration of 50 μM). The fluorescence signal of the mixture was recorded after an incubation time of 30 min at 37 °C. For the kinetic assay, the fluorescence signals were collected after different incubation time with a certain amount of ALP concentration (1 U/mL).

### ALP activity detection in human serum samples

For ALP activity detection in real sample, ALP solutions of different final concentrations were added into the N-CDs/Cu^2+^-PPi system in Tris-HCl buffer solution (pH 7.4) containing 1% diluted human serum. The serum from volunteer was collected by the first affiliated hospital of Zhengzhou University and informed consent was obtained for the use of human serum. All experiments were performed in compliance with the relevant laws and institutional guidelines and approved by Life-Science Ethics Review Committee of Zhengzhou University. NEM was added to the samples to eliminate the RSS in real samples. The following detection procedure was the same as shown in the aforementioned experiment for ALP activity detection in clean Tris-HCl buffer solution.

## Electronic supplementary material

Supplementary Information
