# Supplementary material for: Nitrogen-doped Carbon Dots Mediated Fluorescent on-off Assay for Rapid and Highly Sensitive Pyrophosphate and Alkaline Phosphatase Detection
Source: Sci Rep. 2017 Jul 19;7:5849. doi: 10.1038/s41598-017-06356-z (PMC5517519; doi:10.1038/s41598-017-06356-z)
Supplement: Supplementary file 1 — Supplementary Information [file 41598_2017_6356_MOESM1_ESM.pdf]

Supplementary Information

Nitrogen-doped Carbon Dots Mediated Fluorescent on-off Assay for Rapid and Highly Sensitive Pyrophosphate and Alkaline Phosphatase Detection

Yalei Hu[1], Xin Geng[1], Lin Zhang[1], Zhongming Huang[1], Jia Ge[1,*] and Zhaohui Li[1,2,*]

[1]College of Chemistry and Molecular Engineering, Zhengzhou University, Zhengzhou 450001, P.R. China

[2]Institute of Chemical Biology and Nanomedicine, Hunan University, Changsha 410082, P.R. China

[*] Corresponding authors. Tel.: +86-371-67783007.

E-mail address: zhaohui.li@zzu.edu.cn, jiage0630@hnu.edu.cn

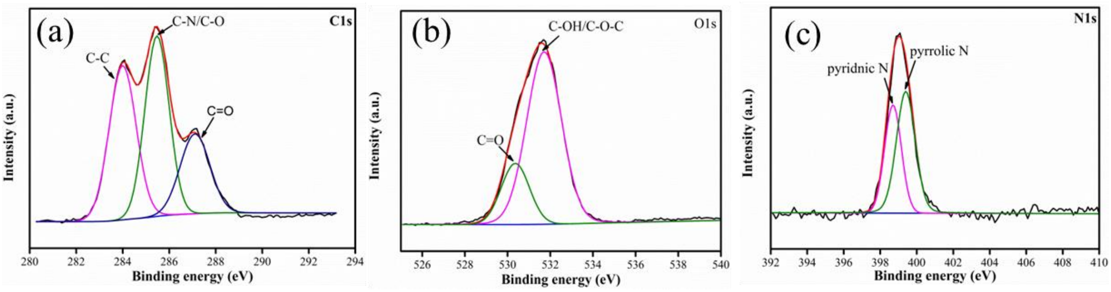

Figure S1. High-resolution N-CDs XPS spectra of C1s (a), O1s (b) and N1s (c).

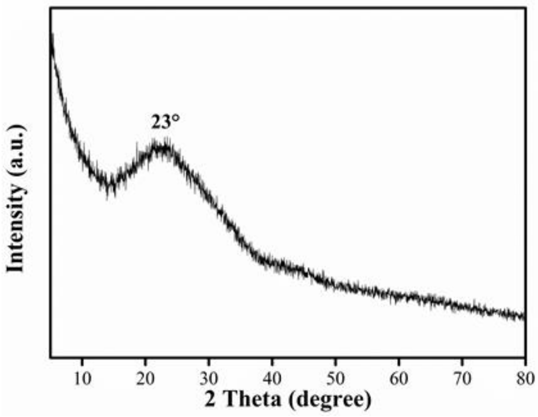

Figure S2. XRD spectrum of N-CDs.

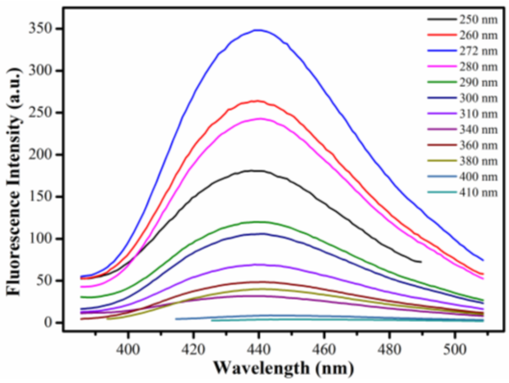

Figure S3. Fluorescence emission spectra of the N-CDs recorded at excitation wavelength from 250 nm to 410 nm.

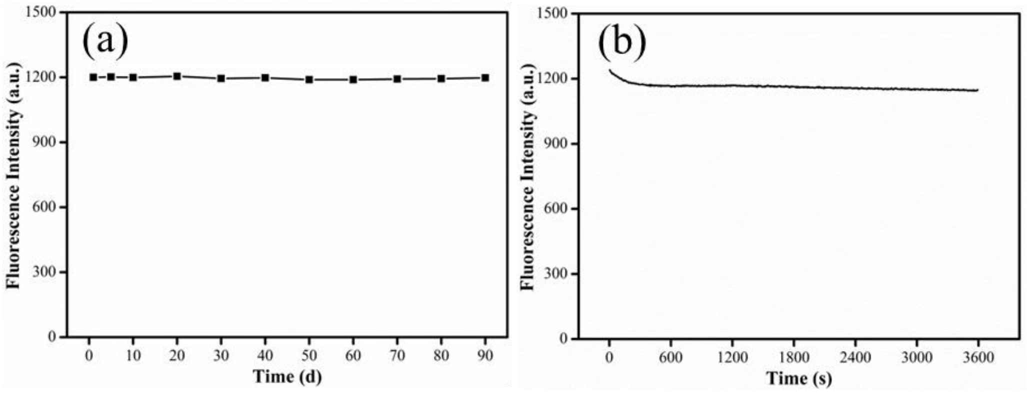

Figure S4. Fluorescence stability of N-CDs in water under room temperature (a) and light illumination (b).

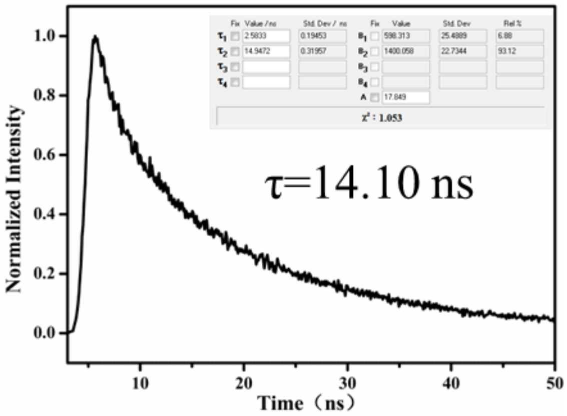

Figure S5. Time-resolved fluorescence decay of N-CDs.

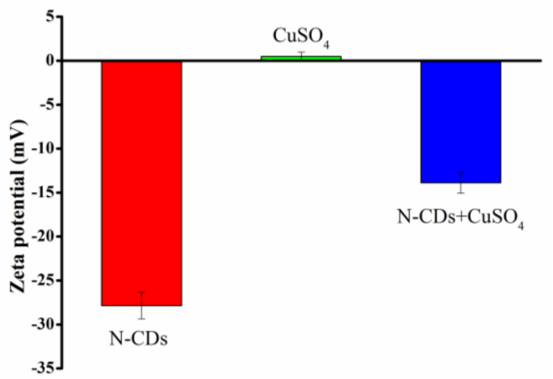

Figure S6. Zeta potentials of CDs solution, $CuSO_4$ solution and CDs/$CuSO_4$ mixture solution.

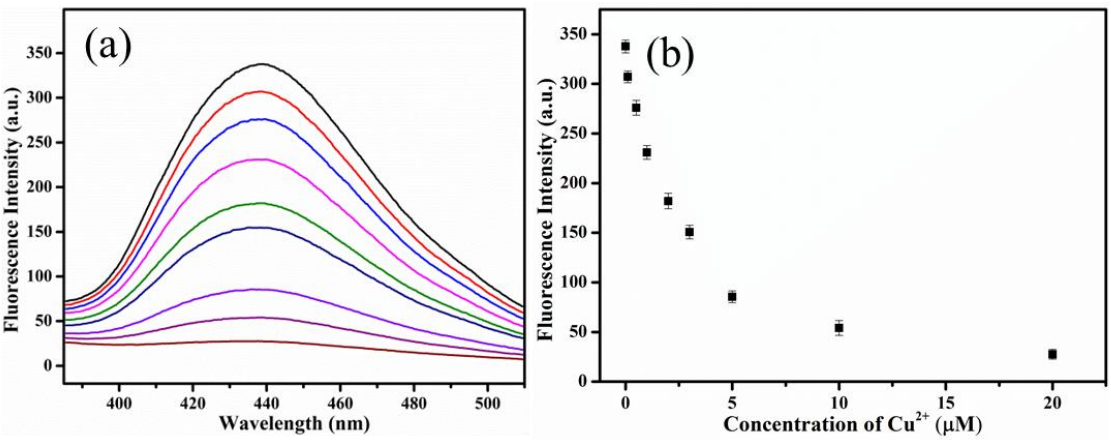

Figure S7. Fluorescence emission spectra (a) and fluorescence responses (b) of N-CDs in the presence of different concentrations of Cu^{2+} (0-20 μM).

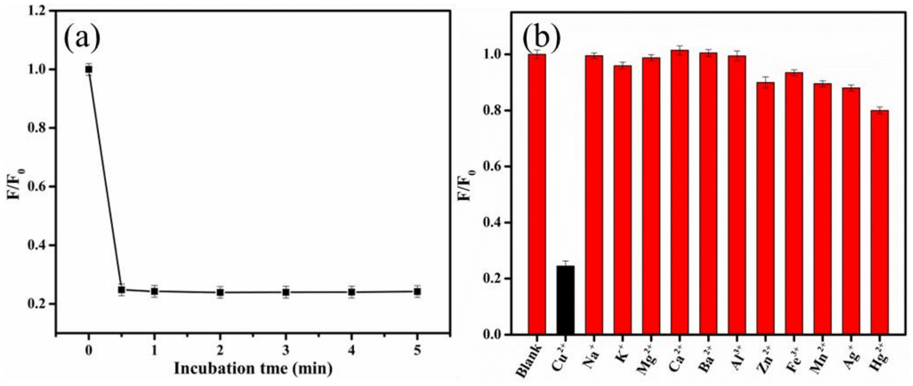

Figure S8. (a) Time-dependent fluorescence intensity of N-CDs in the presence of Cu^{2+}. F_0 is the fluorescence intensity of mere N-CDs and F is the fluorescence intensity of N-CDs/Cu^{2+} with the increasing of incubation time. (b) The effect of different cations on the fluorescence of N-CDs. F_0 is the fluorescence intensity of N-CDs and F is the fluorescence intensity of N-CDs in the presence of different cations. The concentration is 5 μM for Cu^{2+} and 50 μM for other cations.

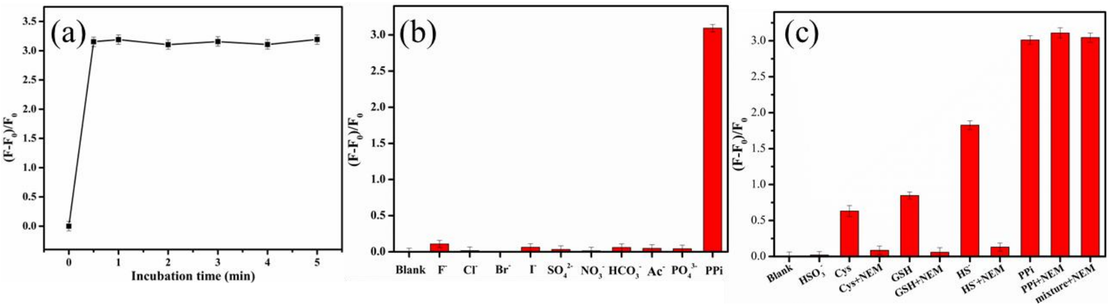

Figure S9. (a) Time-dependent fluorescence intensity of N-CDs/Cu^{2+} in the presence of PPi. F_0 is the fluorescence intensity of N-CDs/Cu^{2+} and F is the fluorescence intensity of N-CDs/Cu^{2+}-PPi system with the increasing of incubation time. (b) The effect of different anions on the fluorescence of N-CDs/Cu^{2+}. F_0 is the fluorescence intensity of N-CDs/Cu^{2+} and F is the fluorescence intensity of N-CDs/Cu^{2+} in the presence of different anions. The concentration is 50 μM for PPi and 500 μM for other anions. (c) Fluorescence response of N-CDs/Cu^{2+} mixture in the presence of 50 μM Cys, GSH, HS^-, HSO_3^- and PPi. The NEM concentrations were 250 μM for single component and 1 mM for the mixture.

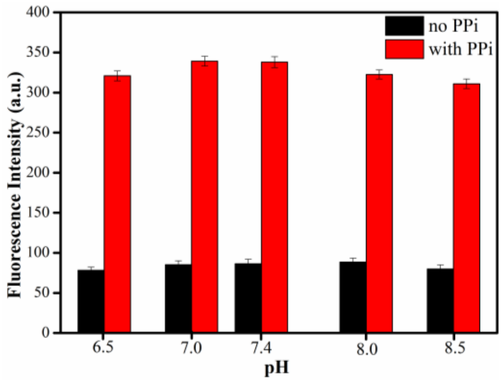

Figure S10. Fluorescence responses of N-CDs/Cu^{2+} mixture in the absence (black bar) and presence (red bar) of PPi at different pH values.

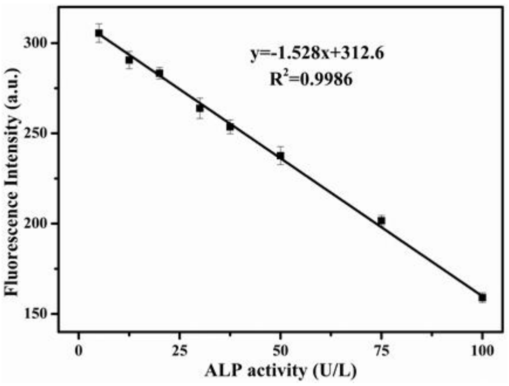

Figure S11. The calibration curve for ALP detection in diluted serum. Error bars were estimated from three replicate measurements.

Table S1. Recovery experiments of ALP in human serum samples.

Samples	Added ALP (U/L)	Detected ALP (U/L)	RSD (%) (n=3)	Recovery (%)
1	12.5	13.0	1.9	104.0
2	30	28.8	1.5	96.0
3	50	49.8	1.2	99.6
